# Reducing co-administration of proton pump inhibitors and antibiotics using a computerized order entry alert and prospective audit and feedback

**DOI:** 10.1186/s12879-016-1679-8

**Published:** 2016-07-22

**Authors:** Christopher E. Kandel, Suzanne Gill, Janine McCready, John Matelski, Jeff E. Powis

**Affiliations:** Department of Medicine, University of Toronto, Toronto, M5S 1A8 Canada; Pharmacy Department, Toronto East General Hospital, Toronto, M4C 3E7 Canada; Division of Infectious Diseases, Toronto East General Hospital, 825 Coxwell Avenue, Toronto, ON M4C 3E7 Canada; Department of General Internal Medicine, Toronto General Hospital, Toronto, M5G 2C4 Canada

**Keywords:** Computerized alerts, Antibiotic stewardship, Proton pump inhibitors, *Clostridium difficile* infection, Prospective audit and feedback

## Abstract

**Background:**

Antibiotics and proton pump inhibitors (PPIs) are associated with *Clostridium difficile* infection (CDI). Both a computer order entry alert to highlight this association as well as antimicrobial stewardship directed prospective audit and feedback represent novel interventions to reduce the co-administration of antibiotics and PPIs among hospitalized patients.

**Methods:**

Consecutive patients admitted to two General Internal Medicine wards from October 1, 2010 until March 31, 2013 at a teaching hospital in Toronto, Ontario, Canada were evaluated. The baseline observation period was followed by the first phase, which involved the creation of a computerized order entry alert that was triggered when either a PPI or an antibiotic was ordered in the presence of the other. The second phase consisted of the introduction of an antibiotic stewardship-initiated prospective audit and feedback strategy. The primary outcome was the co-administration of antibiotics and PPIs during each phase.

**Results:**

This alert led to a significant reduction in the co-administration of antibiotics and PPIs adjusted for month and secular trends, expressed as days of therapy per 100 patient days (4.99 vs. 3.14, *p* < 0.001) The subsequent introduction of the antibiotic stewardship program further reduced the co-administration (3.14 vs. 1.80, *p* <0.001). No change was observed in adjusted monthly CDI rates per 100 patient care days between the baseline and alert cohorts (0.12 vs. 0.12, *p* = 0.99) or the baseline and antibiotic stewardship phases (0.12 vs. 0.13, *p* = 0.97).

**Conclusions:**

Decreasing the co-administration of PPIs and antibiotics can be achieved using a simple automatic alert followed by prospective audit and feedback.

**Electronic supplementary material:**

The online version of this article (doi:10.1186/s12879-016-1679-8) contains supplementary material, which is available to authorized users.

## Background

Hospitalization represents an opportunity to assess medication appropriateness. Proton pump inhibitors (PPIs) are frequently inappropriately used and represent an ideal target for re-evaluation [1, 2]. PPIs increase the risk of *Clostridium difficile* infection (CDI), including both incident episodes as well as recurrences, by approximately 60 % [3, 4]. This elevated risk exists irrespective of whether the infection was acquired in a community or hospital setting [4, 5]. Antibiotics also increase the risk of nosocomial CDI, particularly when prescribed concurrently with a PPI [6–9]. Accordingly, interventions targeting a reduction in the co-administration of PPIs and antibiotics may be beneficial.

Automated alerts activated at the time of computer order entry (CPOE) have been used to reduce medical errors through warnings of impending drug-drug interactions and unrecognized drug allergies. Similar interventions have successfully altered PPI prescribing patterns in the hospital setting and changed antibiotic prescribing patterns [10, 11]. A similar automated strategy may reduce concurrent PPI and antibiotic use, but this has not yet been tested. Extending the purview of antibiotic stewardship programs (ASP), which are successful in lowering antibiotic use in the acute care setting, to identify medications with the potential to increase the harms of antibiotics is another potential intervention to limit co-administration [12].

We evaluated the impact of a real-time automated computer physician order entry (CPOE) alert followed by an ASP-initiated prospective audit and feedback (PAF) strategy on rates of co-administration of PPI’s and antibiotics in a large teaching hospital in Ontario, Canada.

## Methods

### Study design

We prospectively evaluated consecutive patients admitted to two General Internal Medicine wards from October 1, 2010 until March 31, 2013 at a 490-bed teaching hospital in Toronto, Ontario, Canada. The baseline observation interval occurred from October 1, 2010 until September 30, 2011. The first phase, from October 1, 2011 until March 31, 2012, involved the implementation of a CPOE alert that was triggered when either a PPI or an antibiotic was ordered in the presence of the other. This alert highlighted the association of concurrent PPI’s and antibiotics with the increased risk of subsequent CDI. Embedded within the alert was an educational tool that assisted with a risk-benefit analysis of whether to stop a PPI, which for example should be continued in the setting of a recent upper gastrointestinal bleed. The second phase consisted of the introduction of an ASP-initiated PAF strategy, which occurred from April 1, 2012 until March 31, 2013. PAF was triggered from Monday to Friday when an inpatient was prescribed a systemic antimicrobial agent. The aim of the ASP was to provide recommendations regarding the appropriateness of antimicrobial therapy as well as to note the concomitant use of a PPI. Ethics approval was granted from Toronto East General Hospital’s Research Ethics Board.

### Outcome measures

Inpatient orders of antibiotics and PPIs were obtained from the computer order entry system (PowerChart, Cerner Canada, Markham, Canada) and reported as days of therapy (DOT) for each individual medication per 100 inpatient days. One DOT represents the administration of a single medication on a given day irrespective of dosing frequency or strength. Hospital-acquired CDI was confirmed using the Ontario Ministry of Health and Long Term Care case definition with toxin detection by enzyme immunoassay or polymerase chain reaction and presented as a rate over 100 inpatient days [13]. Only loose stool specimens were processed and repeat testing of duplicate specimens within seven days was not permitted. The primary outcome was co-administration of PPI’s and antibiotics. Pre-specified secondary outcomes consisted of CDI rates, PPI use, antibiotic use, and the frequency with which the alert was activated. Only the initial occurrence of the triggered alert for each patient during each hospitalization was used for analysis. Countervailing measures included rates of Gastroenterology consultation and receipt of packed red blood cell transfusion. Transfusions are a marker of acute gastrointestinal bleeding while specialist consultation serves to highlight the possible need for guidance in treating conditions exacerbated by the abrupt cessation of PPI’s.

### Statistical analysis

Generalized linear models were used to analyze systematic differences between the three intervention periods while adjusting for seasonality (monthly effects) and secular trends. In particular, logistic regression was used for the binary outcomes (co-administration, antibiotics, and PPIs), while Poisson regression was used for the count-based outcomes (rates of CDI, packed red blood cell transfusions, and Gastroenterology consultations). For each outcome, we used the Box-Pierce *P*-value against the null hypothesis that the errors from the adjusted model are uncorrelated. Further, we report the adjusted outcome rate for each period (standardized to events per 100 patient care days), and the Wald Test *P*-value against the null hypothesis of ‘no systematic difference’ for each pair of time periods (Baseline-Alert, Baseline-Stewardship, and Alert-Stewardship). For the demographic information collected for each cohort we used a one-way ANOVA and a two-tailed chi-square test to assess for differences between continuous and categorical variables, respectively. The analysis was conducted using R version 3.0.2.

## Results

**Fig. 1:**
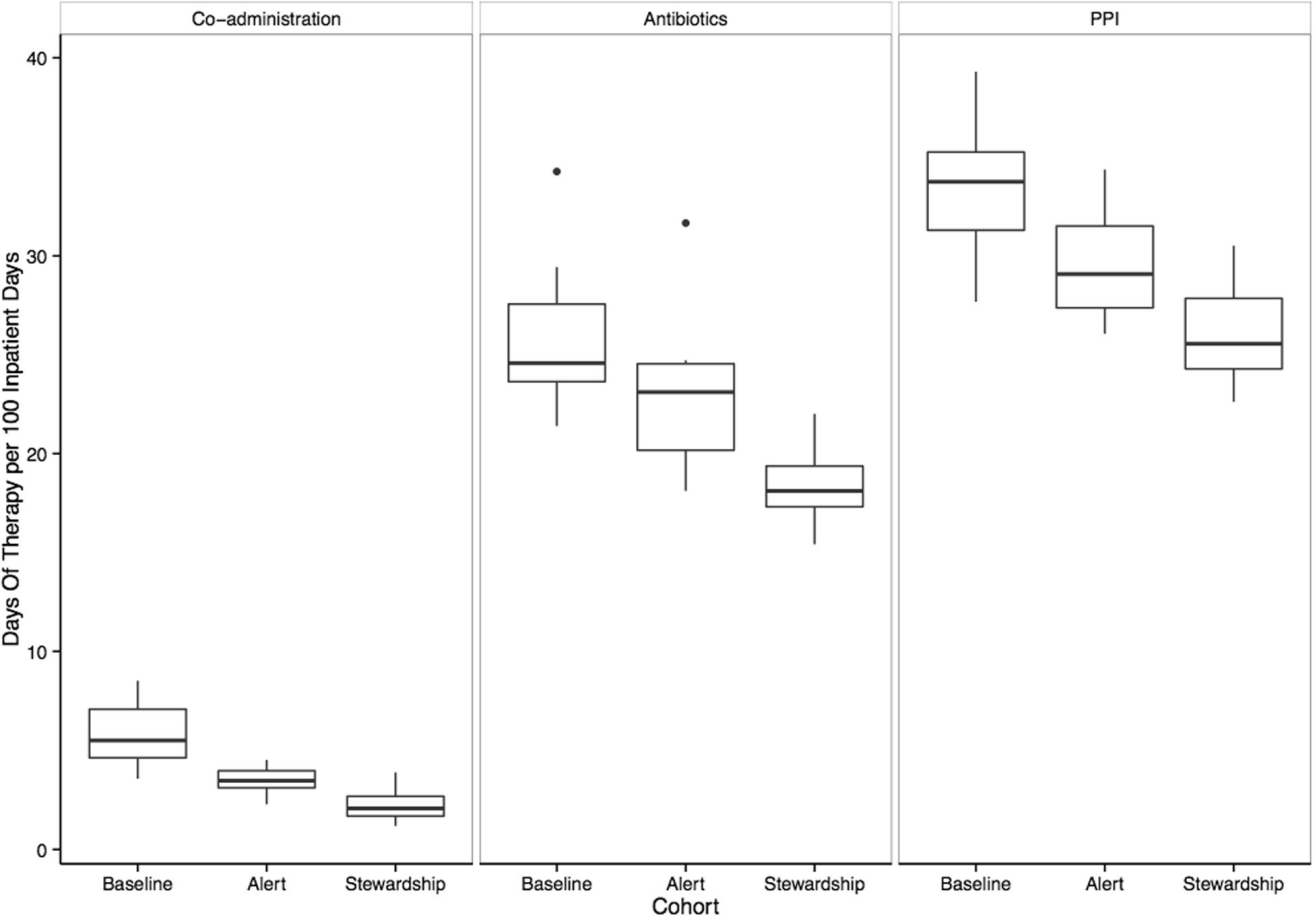
Boxplot of unadjusted average monthly rates of antibiotics, proton pump inhibitors and their co-administration. Rates of co-administration (*left panel*), antibiotics (*middle*) and proton pump inhibitors (*right*) during the baseline, computer alert and antibiotic stewardship program time periods are shown. Rates are calculated as days of therapy (DOT) per 100 inpatient days whereby one DOT represents the administration of a single medication on a given day irrespective of dosing frequency or strength. The vertical lines on either side of the point estimate encapsulate the threshold beyond which data points are considered outliers (represented by dots). PPI = proton pump inhibitor

**Fig. 2:**
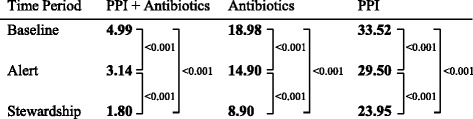
Adjusted average monthly rates of antibiotics, proton pump inhibitors and their co-administration over time. Monthly rates of antibiotics, PPIs, and the co-administration of antibiotics and PPIs are presented as adjusted days of therapy (DOT) per 100 inpatient days whereby one DOT represents the administration of a single medication on a given day irrespective of dosing frequency or strength. *P* values for each time period comparison (baseline-alert, alert-stewardship, and baseline-stewardship) are included. PPI = proton pump inhibitor

A total of 54,005 inpatient days were included (21,542 baseline, 10,763 CPOE, and 21,700 ASP). The three cohorts had similar baseline characteristics and discharge diagnoses, aside from a decrease in the proportion of patients with a respiratory illness among the ASP cohort (Table 1). The unadjusted average monthly co-administration of PPI’s and antibiotics expressed as DOT per 100 patient days varied between the baseline (5.76, 95 % confidence interval 4.78-6.73), alert (3.47, 2.83-4.11), and ASP cohorts (2.22, 1.75-2.69) (Fig. 1). When adjusting for time and month there was a statistically significant decline in the co-administration of PPIs and antibiotics between the baseline and alert phases (4.99 vs. 3.14 DOT per 100 patient days, *p* < 0.001), the baseline and ASP phases (4.99 vs. 1.80, *p* <0.001) and the alert and ASP (3.14 vs. 1.80, *p* <0.001). Antibiotic administration significantly declined over each successive time period from the baseline to the alert and through the initiation of the ASP (18.98 DOT per 100 inpatient days, 14.90, and 8.90, respectively), which was mirrored by a decline in PPIs (33.52, 29.50, and 23.95, respectively) (Fig. 2).

**Table 1:** Baseline demographic characteristics for each study cohort

Variable	Baseline	Computer alert	Antibiotic stewardship	*p* value
Age (mean years ± SD)	71.22 ± 17.64	71.10 ± 17.13	72.47 ± 16.67	0.0212
Male sex (%)	45.92	44.36	45.71	0.6706
Patient care days (mean ± SD)	1795.17 ± 116.64	1793.83 ± 92.16	1808.33 ± 115.74	0.9513
Diagnosis at hospital discharge				
Blood & lymphatic system	56 (2)	28 (2)	51 (2)	0.9324
Circulatory system	216 (9)	119 (10)	179 (8)	0.0581
Digestive system	310 (14)	175 (15)	299 (13)	0.281
Ear, nose, mouth & throat	21 (1)	16 (1)	24 (1)	0.4354
Endocrine system, nutrition & metabolism	143 (6)	66 (6)	115 (5)	0.2956
Hepatobiliary system & pancreas	171 (7)	95 (8)	182 (8)	0.5827
Kidney, urinary tract & male reproductive system	218 (10)	107 (9)	185 (8)	0.3295
Musculoskeletal system & connective tissue	103 (5)	66 (6)	114 (5)	0.2569
Nervous system	234 (10)	123 (11)	276 (12)	0.0588
Respiratory system	187 (8)	75 (7)	89 (4)	<.0001
Skin, subcutaneous tissue & breast	82 (4)	41 (4)	82 (4)	0.9803
Multisystem or unspecified site infections	62 (3)	26 (2)	78 (4)	0.0951
Other	486 (21)	206 (18)	551 (25)	<.0001

Unless otherwise specified, data are no. (%) of patients

*Abbreviations: SD* standard deviation

Other includes burns, diseases of the eye; female reproductive system; mental diseases & disorders; miscellaneous & ungroupable data; other reasons for hospitalization; pregnancy & childbirth; significant trauma, injury, poisoning & toxic effects of drugs

**Fig. 3:**
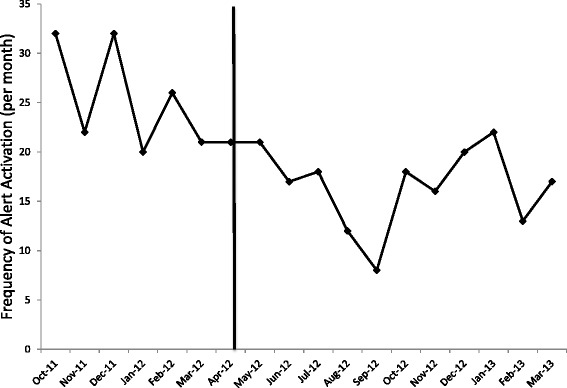
Frequency of computer order entry alert activation. Number of events per month the alert was activated, which occurred when a proton pump inhibitor or antibiotic was ordered in the presence of the other before and after the implementation of the antibiotic stewardship program (depicted by the vertical line). Only the first instance the alert was triggered for each patient during each hospitalization was included

The average monthly frequency of the triggered alert was 25.5 during the alert cohort and 16.9 (*p* = 0.002) during the ASP cohort (Fig. 3). Adjusted monthly CDI rates per 100 inpatient days did not differ between the baseline and alert phases (0.12 vs. 0.12, *p* = 0.99), the baseline and ASP (0.12 vs. 0.13, *p* = 0.97) or between the alert and ASP (0.12 vs. 0.13, *p* = 0.95). Similarly, no changes in adjusted monthly packed red blood cell transfusion rates (1.30 vs. 1.36, *p* = 0.80) or Gastroenterology consultations (2.69 vs. 2.96, *p* = 0.42) between the baseline and alert groups and between the baseline and ASP cohorts (1.30 vs. 1.18, *p* = 0.71 and 2.69 vs. 2.16, *p* = 0.24, respectively) were observed.

## Discussion

We observed a reduction in antibiotic and PPI co-administration following the initiation of both an automated computer order entry alert as well as the introduction of ASP initiated PAF. The alert led to a decrease in PPIs and antibiotics that was immediate and simple to implement. The integration of PPI optimization to the responsibilities of an ASP afforded further reductions in co-administration beyond that realized from the alert and allowed for sustainability of the effect.

Automated alerts can alter PPI prescribing patterns and may have prompted clinicians to reconsider the utility of PPI’s in the high-risk setting of prescribing antibiotics [6, 10]. The alert may have engendered an additional evaluation of the risk-benefit ratio of prescribing antibiotics that cumulatively led to the reduction in antibiotic use observed. Lowering overall antibiotic use has been associated with a reduced incidence of nosocomial CDI while it is unclear whether a similar benefit occurs with PPIs [14]. The decrease in co-administration was not associated with a reduction in CDI rates, however the low overall event rate limited statistical power. Antibiotic classes variably affect CDI risk yet in the hospital setting cumulative antibiotic exposure is an independent risk factor for CDI making it imperative to target all antibiotics and not restrict the alert to only those associated with an elevated risk [15, 16]. The abrupt cessation of a PPI results in rebound acid hypersecretion that can potentially exert a protective effect against contracting *C. difficile* through an increase in gastric acidity [17]. The reduction in PPIs did not result in a change in the rates of red blood cell transfusion or Gasteroenterology consultation, both of which are relatively crude markers of harm

The introduction of PAF by the ASP was followed by further reduction in antibiotic and PPI co-administration compared to both the baseline and the alert cohorts. The reduction in antibiotic administration is commensurate with the effectiveness of ASPs in the inpatient setting [12]. Additionally, the ASP led to a reduction in PPI administration, suggesting that it is feasible to expand the purview of ASPs beyond solely antimicrobials. The decrease in alert frequency following the introduction of the ASP suggests a persistent effect on prescribing practices and cumulative benefit through enacting culture change mediated by the ASP. Importantly, the ASP led to a sustained reduction in the co-administration of PPIs and antibiotics for an additional 12 months, obviating the loss of motivation to follow alert recommendations commonly responsible for the decreased effectiveness of electronic alerts that occurs over time [18]. ASPs should evolve to encompass more than assessing antibiotic appropriateness, as they are ideally situated to provide advice towards attenuating the potential harms of antibiotics, such as decreasing PPI and antibiotic co-administration.

There are some limitations that merit emphasis. Firstly, the short duration of observation may not accurately reflect the potential benefits observed. Although we adjusted for seasonality in our analysis, the total duration of the study was short and the CDI event rate was low. The low CDI event rate also precluded analyzing risk factors for CDI, including patient-related characteristics such as age and underlying comorbidities, which may have contributed to the absence of statistically significant differences in CDI rates [19]. Secondly, the single site and pre-existing comprehensive ASP, which is activated upon the prescribing of an antibiotic for all inpatients, may affect external validity. Thirdly, robust measures of harm could not be collected and may be underappreciated, especially in light of the short study interval. Fourthly, outcomes were restricted to in-hospital events, which may underestimate the benefit of the interventions.

## Conclusions

Reducing the co-administration of PPI’s and antibiotics can be achieved with an automatic computerized alert and implementation of an ASP. The computer order entry alert facilitates enhanced awareness of drug interactions that may curtail inappropriate medication use. ASP mediated PAF implemented after the computer order alert provides an opportunity to consolidate alert-mediated changes in prescribing practices through cultural change allowing for sustainability and attenuating alert fatigue. Reproducing this study using a larger sample size with a longer duration of follow up will serve to test whether a reduction in PPI-antibiotic co-administration can be sustained, the independent impact of computer order alerts and ASP and whether the reduction in co-administration lowers CDI rates.

## Abbreviations

ASP, antibiotic stewardship program; CDI, *Clostridium difficile* infection; CPOE, computer order entry; DOT, days of therapy; PAF, prospective audit and feedback; PPI, proton pump inhibitor

## Additional file

Additional file 1: Table S1. Raw monthly data for the primary and secondary outcomes. (XLSX 11 kb)
